# QSAR-based molecular signatures of prenylated (iso)flavonoids underlying antimicrobial potency against and membrane-disruption in Gram positive and Gram negative bacteria

**DOI:** 10.1038/s41598-018-27545-4

**Published:** 2018-06-18

**Authors:** Carla Araya-Cloutier, Jean-Paul Vincken, Milou G. M. van de Schans, Jos Hageman, Gijs Schaftenaar, Heidy M. W. den Besten, Harry Gruppen

**Affiliations:** 1 Laboratory of Food Chemistry, Wageningen University & Research, Wageningen, The Netherlands; 2 RIKILT – Wageningen University & Research, Wageningen, The Netherlands; 3 Biometris, Applied Statistics, Wageningen University & Research, Wageningen, The Netherlands; 4 Nijmegen Centre for Molecular Sciences, Radboud University Medical Centre, Nijmegen, The Netherlands; 5 Laboratory of Food Microbiology, Wageningen University & Research, Wageningen, The Netherlands

## Abstract

Prenylated flavonoids and isoflavonoids are phytochemicals with remarkable antibacterial activity. In this study, 30 prenylated (iso)flavonoids were tested against *Listeria monocytogenes* and *Escherichia coli* (the latter in combination with an efflux pump inhibitor). Minimum inhibitory concentrations of the most active compounds ranged between 6.3–15.0 µg/mL. Quantitative structure-activity relationships (QSAR) analysis was performed and linear regression models were proposed with R^2^ between 0.77–0.80, average R^2^_m_ between 0.70–0.75, Q^2^_LOO_ between 0.66–0.69, and relatively low amount of descriptors. Shape descriptors (related to flexibility and globularity), together with hydrophilic/hydrophobic volume and surface area descriptors, were identified as important molecular characteristics related to activity. A 3D pharmacophore model explaining the effect of the prenyl position on the activity of compounds was developed for each bacterium. These models predicted active compounds with an accuracy of 71–88%. With regard to the mode of action, good antibacterial prenylated (iso)flavonoids with low relative hydrophobic surface area caused remarkable membrane permeabilization, whereas those with higher relative hydrophobic surface area did not. Based on the QSAR and membrane permeabilization studies, the mode of action of antibacterial prenylated (iso)flavonoids was putatively rationalized.

## Introduction

Interest in natural compounds for antimicrobial discovery has increased during the last decade due to the rise in highly resistant bacteria^1^. Natural products are a potentially rich source of compounds for antimicrobial discovery and development, as they usually have a high degree of stereochemistry with an extensive variety of (ring) scaffolds and generally low toxicity^2,3^.

In the Fabaceae plant family, prenylated phenolic compounds have been identified as promising antimicrobials in extracts from elicited seedlings^4,5^. These phenolic compounds mainly belong to the flavonoid (2-phenyl benzopyrans), isoflavonoid (3-phenyl benzopyrans) and stilbenoid (1,2-diphenyl ethylene) classes. Besides the skeleton, they can differ in configuration and position of prenylation. Prenyl groups can be attached to a phenolic compound as a chain (3,3-dimethylallyl substituent) or as a five- or six-membered ring after undergoing enzymatic cyclisation with an *ortho*-phenolic hydroxyl group^6^. Prenylation has been shown to increase the antibacterial activity of phenolic compounds^7^, partly because hydrophobicity increases. Thereby, the partitioning to biological targets, such as membranes, increases^8^.

Structure-antibacterial activity relationships (SAR) of prenylated (iso)flavonoids have been obtained by comparing the activity data and structures of a few analogues (usually ≤ 10 compounds)^9,10^. Studies have focused on describing the substituents and positions within a particular phenolic skeleton that provide high or low antibacterial activity^11,12^. The antibacterial activity of prenylated and non-prenylated (iso)flavonoids from licorice against methicillin-resistant *Staphylococcus aureus* (MRSA) and Gram negative bacteria has been studied^13^. None of the compounds were active against the Gram negative bacteria (minimum inhibitory concentration (MIC) >128 µg/mL), but some were very active against MRSA, especially diprenylated (iso)flavonoids (MIC 8.0 µg/mL). It was also observed that the presence of a D-ring in a coumestan was detrimental for the activity, the effect of which was attributed to the rigidity of the skeleton. In contrast, a diprenylated 6a,11a-pterocarpene and a monoprenylated pterocarpan, both having rigid skeletons, were the most antibacterial compounds against MRSA (MIC 6.3 µg/mL for both)^14^. In another study^15^, a diprenylated pterocarpan and diprenylated isoflavan were found to be very active (MIC 3.1–6.3 µg/mL) against vancomycin-resistant enterococci, whereas a diprenylated isoflavanone was not. Further studies showed that the position of the prenyl group(s) differently affected the antibacterial activity of different subclasses of (iso)flavonoids^16,17^. For flavanones, prenylation at *C*8 provided a higher antibacterial activity (i.e. MIC 12.5–25 µg/mL) than prenylation at *C*6 (MIC >100 µg/mL)^18^, whereas for isoflavones the opposite result was found^10^. Regarding other substituents, the presence of hydroxyl groups appeared to be essential for antibacterial activity of prenylated (iso)flavonoids^7^. In contrast, the influence of methoxyl groups was more ambiguous, with reduced activity in some isoflavonoids^14,19^, and enhanced activity in others^12,17,20^.

Increase in hydrophobicity by the presence of prenyl groups has been the traditional explanation given for the increase in antibacterial activity observed upon prenylation of (iso)flavonioids^8^. However, not all prenylated (iso)flavonoids have shown antibacterial activity^18,21^, highlighting the importance of other, yet to be defined, molecular characteristics for antibacterial activity. Furthermore, it has not been investigated in any large detail how differences in the overall molecular configuration relate to the mode of action. In this context, *in silico* approaches, like quantitative SAR (QSAR)^22^ and pharmacophore modelling^23^, can help to elucidate the physicochemical properties important for the antibacterial activity and mode of action. Despite numerous descriptive SAR studies of prenylated (iso)flavonoids, frequently with relatively few compounds, no QSAR analysis of prenylated (iso)flavonoids as antibacterials has been performed so far.

**Figure 1:**
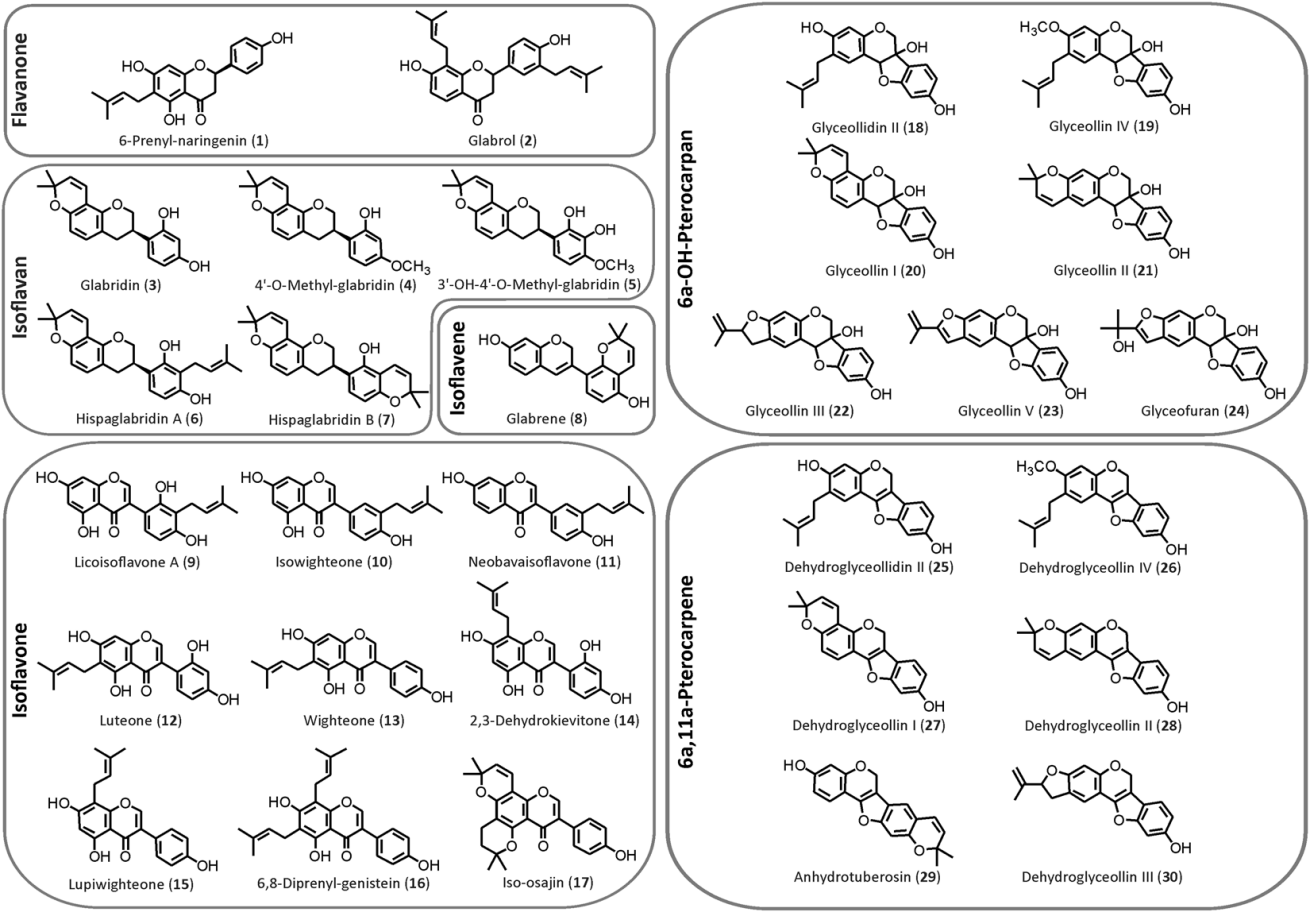
Overview of the structures of the prenylated (iso)flavonoids, and subclass to which they belong, tested in this study.

In this study we report on the application of QSAR analysis to elucidate the main molecular characteristics of prenylated (iso)flavonoids as antibacterials. For this, we used the Gram positive *Listeria monocytogenes* and the Gram negative *Escherichia coli* as model organisms. We previously showed that intrinsic resistance in *E*. *coli* can be overcome by inhibiting the efflux pump systems^5^. Consequently, we here report the antibacterial activity of prenylated (iso)flavonoids against *E*. *coli* in combination with an efflux pump inhibitor. We tested 30 mono- and diprenylated (iso)flavonoids (Fig. 1), belonging to 6 different (iso)flavonoid subclasses, prenylated at different positions of the main skeleton and with chain, pyran or furan prenyl configurations. The majority of these molecules has never been tested before against *L*. *monocytogenes*, or against *E*. *coli* in the presence of an efflux pump inhibitor. We determined the minimum inhibitory and bactericidal concentrations of all compounds, and analyzed the membrane permeabilization capacity of the antibacterial compounds.

The main goals of this study were (i) to develop a multiple linear regression model with high predictive capabilities for the activity of other prenylated (iso)flavonoids by using a QSAR approach, and (ii) to understand the mode of action of prenylated (iso)flavonoids against Gram positive and Gram negative bacteria by linking the QSAR findings to the proposed mode of action of prenylated (iso)flavonoids, i.e. disruption of membrane integrity^5,24–26^.

## Results

Table 1 shows the MICs and MBCs determined for each compound against each bacterium. Diprenylated glabrol (**2**) and 6,8-diprenylgenistein (**16**) were very good antibacterials against *L*. *monocytogenes* with both MIC and MBC values of 6.3 µg/mL. *E*.*coli* was not susceptible to any of the tested (iso)flavonoids up to 50 µg/mL. When the efflux pumps were inhibited by PAβN, *E*. *coli* became susceptible to a similar extent as *L*. *monocytogenes* (control samples with *E*. *coli* demonstrated no growth inhibition by PaβN alone, data not shown). In this way, monoprenylated glabridin and luteone showed very good antibacterial activity against *E*. *coli* with MICs of 10 µg/mL and MBCs of 15.0 µg/mL.

**Table 1:** Antibacterial activity of prenylated (iso)flavonoids.

No.	Name compound	*L*. *monocytogenes*		*E*. *coli* + EPI	
		MIC	MBC	MIC	MBC
**Flavanone**					
1	6-Prenyl-naringenin	>50	>50	25	25
2	Glabrol	6.3	6.3	>50	>50
**Isoflavan**					
3	Glabridin	15	20	10	15
4	4′-*O*-methyl-glabridin	10	12.5	20	20
5	3′-OH-4′-*O*-methyl-glabridin	12.5	25	>50	>50
6	Hispaglabridin A	15	25	>50	>50
7	Hispaglabridin B	10	15	>50	>50
**Isoflavene**					
8	Glabrene	35	50	50	50
**Isoflavone**					
9	Licoisoflavone A	35	50	20	25
10	Isowighteone	20	20	25	25
11	Neobavaisoflavone	50	50	25	25
12	Luteone	20	50	10	15
13	Wighteone	10	15	15	15
14	2,3-Dehydrokievitone	>50	>50	>50	>50
15	Lupiwighteone	>50	>50	>50	>50
16	6,8-Diprenyl-genistein	6.3	6.3	>50	>50
17	Iso-osajin	>50	>50	>50	>50
**6a-OH-Pterocarpan**					
18	Glyceollidin II	>50	>50	>50	>50
19	Glyceollin IV	50	>50	50	>50
20	Glyceollin I	>50	>50	>50	>50
21	Glyceollin II	>50	>50	>50	>50
22	Glyceollin III	>50	>50	>50	>50
23	Glyceollin V	>50	>50	>50	>50
24	Glyceofuran	>50	>50	>50	>50
**6a**,**11a-Pterocarpene**					
25	Dehydroglyceollidin II	12.5	15	50	>50
26	Dehydroglyceollin IV	25	35	>50	>50
27	Dehydroglyceollin I	20	20	>50	>50
28	Dehydroglyceollin II	20	25	>50	>50
29	Anhydrotuberosin	>50	>50	n.t.^*c*^	n.t.
30	Dehydroglyceollin III	>50	>50	>50	>50

Minimum inhibitory concentration (MIC, µg/mL) and minimum bactericidal concentration (MBC, µg/mL) of prenylated (iso)flavonoids against *L*. *monocytogenes* EGD-e and against *E*. *coli* K12, the latter one in the presence of efflux pump inhibitor PaβN (48 µM). No antibacterial activity was observed against *E*. *coli* without the efflux pump inhibitor (MIC >50 µg/mL). n.t. Not tested.

### Effect of (iso)flavonoid subclass

**Figure 2:**
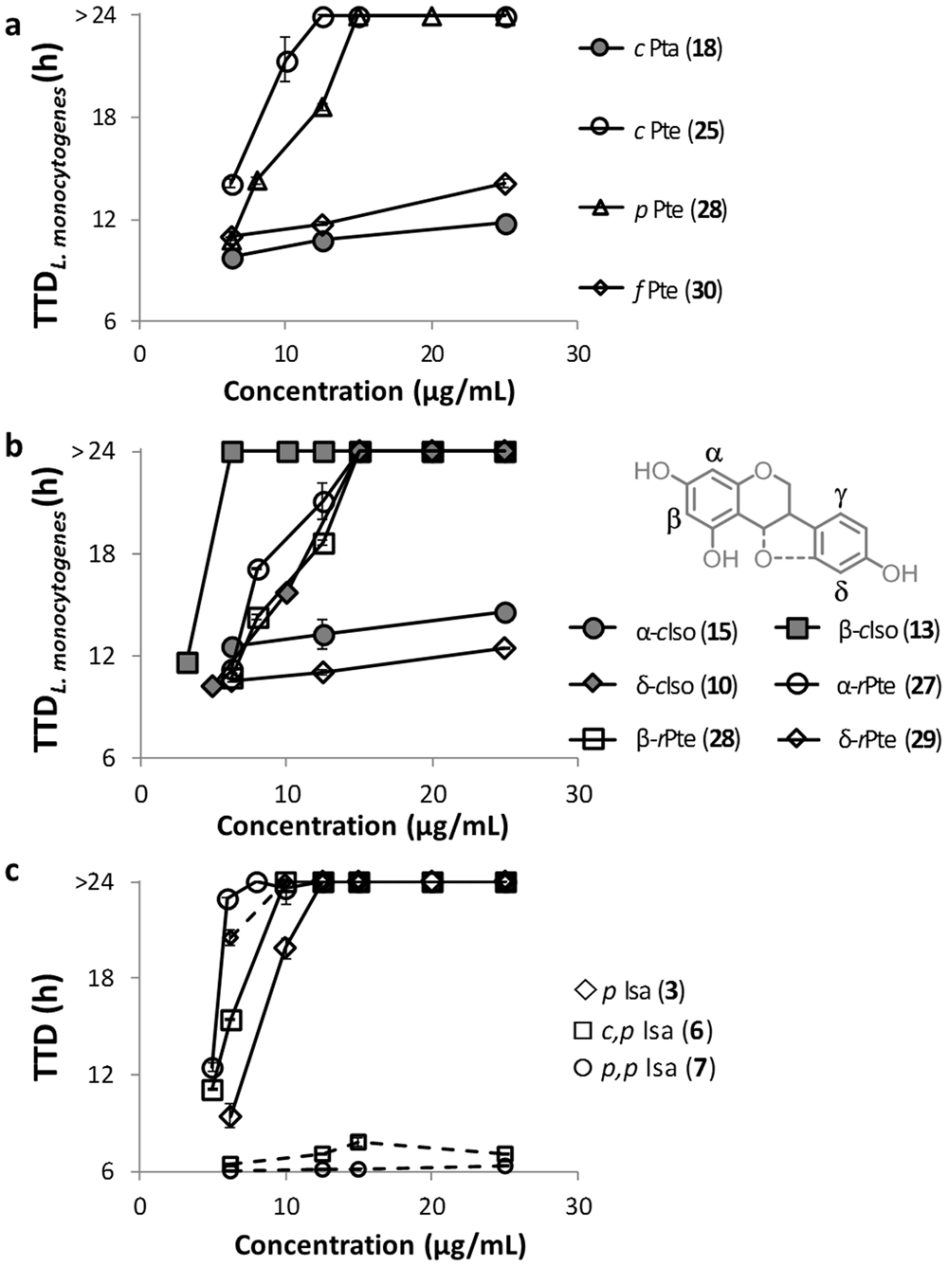
Time to detection (TTD) of bacterial growth upon exposure to prenylated (iso)flavonoids. (**a**) TTD of *L*. *monocytogenes* growth in the presence of different prenylated 6a-hydroxy-pterocarpans (glyceollidin II, **18**) and 6a,11a-pterocarpenes (dehydroglyceollidin II, **25**; dehydroglyceollin II, **28;** dehydroglyceollin III, **30**). (**b**) TTD of *L*. *monocytogenes* growth in the presence of chain prenylated isoflavones (closed symbols): isowighteone (**10**), wighteone (**13**), lupiwighteone (**15**); and ring-closed prenylated pterocarpenes (open symbols): dehydroglyceollin I (**27**), dehydroglyceollin II (**28**), anhydrotuberosin (**29**). Prenyl positions are annotated based on the inset. (**c**) TTD of *L*. *monocytogenes* (solid line) and *E*. *coli* (with EPI, dashed line) growth in the presence of different prenylated isoflavans: glabridin (**3**), hispaglabridin A (**6**) and hispaglabridin B (**7**). Error bars represent the standard deviation of two biologically independent reproductions. TTD of control cells (i.e. cells not exposed to prenylated compounds) was 11.1 ± 2.9 h for *L*. *monocytogenes* and 6.9 ± 0.13 for *E*. *coli*.

In general, isoflavans and isoflavones were among the most active compounds against both *L*. *monocytogenes and E*. *coli* (Table 1). Interestingly, most 6a-hydroxy-pterocarpans did not show antibacterial activity up to the highest concentration tested (MIC >50 µg/mL) against the two target bacteria, whereas 6a,11a-pterocarpenes were active against *L*. *monocytogenes*. Figure 2a illustrates the inhibition of growth of *L*. *monocytogenes*, when exposed to a chain prenylated 6a-hydroxy-pterocarpan (*c*Pta, **18**) and its equivalent chain prenylated 6a,11a-pterocarpene (*c*Pte, **25**) (the higher the TTD, the stronger the inhibition with a maximum of 24 h). As observed, the dehydration and formation of a double bond at position 6a in dehydroglyceollidin II (**25**) enhanced the antibacterial activity in comparison with glyceollidin II (**18**).

### Effect of the configuration of prenyl group

Figure 2a also compares the inhibitory activity of three 6a,11a-pterocarpenes sharing the same structure but different configuration of prenyl group. The chain prenylated 6a,11a-pterocarpene (*c*Pte, **25**) inhibited the growth of the bacteria at a minimum concentration of 12.5 µg/mL (Fig. 2a). The pyran prenylated 6a,11a-pterocarpene (*p*Pte, **28**) was the next most active compound with a MIC of 20.0 µg/mL. Last, the furan prenylated 6a,11a-pterocarpene (*f*Pte, **30**) did not inhibit the growth at any concentration tested. Also, none of the furan prenylated compounds (**22–24**, **30**) showed antibacterial activity against the target bacteria (Table 1).

### Effect of the position of the prenyl group

Concerning the (iso)flavonoid skeletons, there are four main positions of *C*-prenylation: *C*6, *C*8, *C*3’ and *C*5’ (or the equivalents *C*2, *C*4, *C*8 and *C*10 in 6a-hydroxy-pterocarpans and 6a,11a-pterocarpenes). In order to compare different subclasses of (iso)flavonoids, the Greek numbering system of Simons *et al*^6^. was adopted, which is illustrated by the isoflavonoid skeleton in Fig. 2b. The position of the prenyl group clearly affected the antibacterial activity of these compounds. Lupiwighteone, chain prenylated at the α position (α-*c*Iso, **15**), showed no inhibition towards *L*. *monocytogenes*, whereas β-prenylated wighteone (β-*c*Iso, **13**) had high antibacterial activity, followed by δ-prenylated isowighteone (δ-*c*Iso, **10**). For 6a,11a-pterocarpenes, activity was similar for α- and β-prenylated compounds, i.e. dehydroglyceollin I (α-*r*Pte, **27)** and II (β-*r*Pte, **28**), whereas the δ-prenylated 6a,11a-pterocarpene, i.e. anhydrotuberosin (δ-*r*Pte, **29**), showed the lowest growth inhibition.

### Effect of the number of prenyl groups

In Fig. 2c the growth inhibition of *L*. *monocytogenes* (solid line) and *E*. *coli* in the presence of the EPI (dashed line) by mono- and diprenylated isoflavans (Isa) is illustrated. Except for iso-osajin (**17**), diprenylated (iso)flavonoids were among the most active ones against the Gram positive bacterium (MIC ≤ 15 µg/mL). Diprenylated hispaglabridin A (*c*,*p*Isa, **6**) and B (*p*,*p*Isa, **7**) inhibited bacterial growth faster than monoprenylated glabridin (*p*Isa, **3**). Opposite results were found for the Gram negative bacterium. Diprenylated (iso)flavonoids were not effective antibacterials against *E*.*coli* (Table 1), whereas monoprenylated compounds, including glabridin (**3**), were very active against this bacterium (MIC ≤ 15 µg/mL).

### Effect of other substituents

The number of hydroxyl groups influenced the antibacterial activity of prenylated isoflavones. The presence of 3 OH groups in the isoflavone skeleton as in isowighteone (**10**) was more effective against the Gram positive bacteria (MIC 20 µg/mL) than the presence of 4 OH groups as in licoisoflavone A (**9**, MIC 35 µg/mL) or than the presence of 2 OH groups as in neobavaisoflavone (**11**, MIC 50 µg/mL). For *E*. *coli*, the prenylated isoflavone with 4 OH groups (**9**, MIC 20 µg/mL) was slightly better antibacterial than prenylated isoflavones with 3 (**10)** or 2 OH groups (**11**, MIC 25 µg/mL). With regard to *O*-methylation, we found no clear effect on the antibacterial activity of prenylated (iso)flavonoids against *L*. *monocytogenes*. In the case of *E*. *coli*, *O*-methylation was detrimental for activity, as in dehydroglyceollidin II (**25**, MIC 50 µg/mL) in comparison with *O*-methylated dehydroglyceollin IV (**26**, MIC >50 µg/mL), or as in glabridin (**3**, MIC 15 µg/mL) in comparison with 4’-*O*-methyl-glabridin (**4**, MIC 20 µg/mL).

### QSAR analysis

QSAR of prenylated (iso)flavonoids was carried out to interpret the structural features responsible for their antibacterial activity against Gram positive *L*. *monocytogenes* and Gram negative *E*. *coli* (in combination with an efflux pump inhibitor). A genetic algorithm (GA) was used to explore which molecular descriptors were best able to predict the antibacterial activity using ordinary least square (OLS) regression. GA is an optimization technique that explores the descriptor space simultaneously by a population of candidate solutions (models), in which solutions compete and recombine^27^. Since the starting point of a GA run is always different, every GA run was repeated 5 times for a fixed subset of descriptors (varied from 3 to 8 descriptors). The best OLS models obtained per number of descriptor are summarized in Table 2 (criteria for selecting the best models are specified in Materials and Methods section). The descriptors used in the best GA-generated OLS models are specified in Table 3.

**Table 2:** Statistical performance of the best OLS models obtained using GA-selection of variables for predicting antibacterial activity of prenylated (iso)flavonoids against *L*. *monocytogenes* and *E*. *coli*.

Bacteria	k	*p*-value	R^2^	R^2^_adj_	SE	Q^2^_LOO_	Q^2^_LOO_adj_	VIF_max_
*L*.*monocytogenes*	3	5.2e-06	0.6431	0.6020	0.2807	0.5537	0.5022	1.957
	4	3.2e-05	0.6328	0.574	0.2904	0.6685	0.6155	4.844
	**5**	**4**.**6e-07**	**0**.**7735**	**0**.**7263**	**0**.**2328**	**0**.**6566**	**0**.**5850**	**1**.**592**
	6	4.9e-07	0.8007	0.7487	0.2230	0.6821	0.5991	3.413
	7	3.8e-06	0.7886	0.7213	0.2349	0.7044	0.6103	4.732
	8	1.5e-05	0.7866	0.7053	0.2415	0.6788	0.5564	4.567
*E*. *coli*	3	2.2e-05	0.6140	0.5677	0.2233	0.4955	0.4373	1.745
	4	7.8e-06	0.6881	0.6361	0.2049	0.5736	0.5054	2.251
	**5**	**1**.**8e-07**	**0**.**8047**	**0**.**7623**	**0**.**1656**	**0**.**6921**	**0**.**6279**	**4**.**143**
	6	2.0e-06	0.7879	0.7301	0.1765	0.6620	0.5738	2.317
	7	1.2e-07	0.8597	0.813	0.1469	0.7569	0.6795	4.358
	8	7.0e-07	0.8564	0.7989	0.1523	0.7356	0.6349	3.999

k: number of descriptors; R^2^: coefficient of determination; R^2^_adj_: adjusted R^2^; SE: standard error of residuals; Q^2^_LOO_: leave-one-out cross-validated coefficient of determination; Q2_LOO_adj_: adjusted Q^2^_LOO_; VIF_max_: maximum variance inflation factor. The finally chosen OLS models are shown in bold.

**Table 3:** Descriptors present in the best OLS regression models obtained for predicting the antibacterial activity against *L*. *monocytogenes* and *E*. *coli*.

*L. monocytogenes*				*E. coli*			
Descriptor	F	*p*-value	Sign	Descriptor	F	*p*-value	Sign
a_ICM	1	1.8E^−06^	−	a_acc	2	4.8E^−06^–0.0149	−
ast_violation	1	0.0019	+	a_hyd	2	8.0E^−06^–8.5E^−05^	−
b_rotN	1	0.0185	+	b_count	1	8.4E^−07^	−
dens	1	0.0326	−	b_single	1	0.0006	−
diameter	1	0.0088	−	logP_o/w_	1	0.0055	−
glob	1	0.1308	+	npr1	2	0.0003–0.0018	−
h_ema	1	8.1E^−05^	−	PEOE_VSA+0	1	0.0139	−
KierA3	2	1.2–2.2E^−05^	+	petitjeanSC	1	0.0028	−
rsynth	5	2.1E^−05^–0.0085	+	rgyr	2	1.1E^−05^–0.0001	+
vdw_area	1	0.0019	+	RPC+	1	0.0012	+
vsurf_A	2	0.0015–0.0027	−	SlogP_VSA0	1	0.2686	−
vsurf_CP	1	0.1804	+	std_dim3	6	8.6E^−08^–0.0002	+
vsurf_CW2	1	0.6293	−	vsurf_A	1	0.0011	−
vsurf_CW6	3	0.0045–0.0191	+	vsurf_CW1	1	0.0808	+
vsurf_DD12	3	0.0001–0.0049	−	vsurf_EDmin1	1	0.0076	+
vsurf_DW12	1	0.1013	−	vsurf_IW1	1	0.0002	+
vsurf_ID8	1	0.0111	+	vsurf_IW2	3	5.1E^−06^–7.3E^−05^	+
vsurf_IW4	1	0.0078	−	vsurf_IW4	1	0.0004	−
vsurf_IW5	1	0.0484	+	vsurf_IW5	1	0.0023	−
vsurf_IW6	1	0.1961	+	vsurf_IW6	1	0.0005	−
vsurf_IW7	1	0.1350	−	vsurf_W7	1	0.0190	−
vsurf_W4	1	0.0114	+	zagreb	1	0.0002	−
vsurf_W6	1	0.0004	+				

F: selection frequency in best GA-generated models; *p*-value: significance of descriptor coefficient in the models; Sign: sign of descriptor coefficient. Descriptor meaning can be found in Supplementary Table S3.

#### *L. monocytogenes* models

The top most frequently used descriptors (i.e. those with a frequency factor larger than 1 in Table 3) in the models of *L*.*monocytogenes* were related to the fraction of heavy atoms (*rsynth*, *p*-value 0.008), molecular branching (*KierA3*, *p*-value 2 × 10^−5^), the relative hydrophilic surface (*vsurf_CW6*, *p*-value 0.002), distribution of hydrophilic/hydrophobic regions (*vsurf_A*, *p*-value 0.003), and the location of hydrophobic interacting atoms or groups (*vsurf_DD1*2, *p*-value 0.005).

Additionally, considering the sign (positive/negative) of the coefficient estimates of the descriptors in Table 3, the molecular properties that are important for antibacterial activity of prenylated (iso)flavonoids might be predicted. Specifically, shape related descriptors, i.e. molecular branching (*KierA3*, top descriptor), number of rotational bonds (*b_rotN*) and globularity (*glob*) were positively correlated with antibacterial activity, whereas density (*dens*), atom information content (*a_ICM*) and diameter (*diameter*) were negatively correlated with activity. This gave an indication that small flexible globular molecules were better for antibacterial activity against *L*. *monocytogenes* than large flat molecules. The hydrophilic surface (*vsurf_CW6*, top descriptor) and volume (*vsurf_W*) were positively correlated with activity, but having separate hydrophilic and hydrophobic regions within the molecule seems to be detrimental for activity, as the amphiphilic moment (*vsurf_A*, top descriptor) was negatively correlated with activity. The (positive/negative) contribution of other (top) descriptors (e.g. *rsynth*, *vsurf_DD*, *vsurf_IW*) in relation to antibacterial activity remains difficult to interpret and needs further study.

#### *E. coli* models

The top most frequently used descriptors (i.e. those with a frequency factor larger than 1 in Table 3) in the models of *E*. *coli* were related to molecular shape (*std_dim3*, *p*-value 0.0002; *npr1*, *p*-value 0.002), the distribution of hydrophilic regions (*vsurf_IW*2, *p*-value 7 × 10^−5^), the number of hydrogen bond acceptors (*a_acc*, *p*-value 0.01), the number of hydrophobic atoms (*a_hyd*, *p*-value 8 × 10^−5^), and molecular flexibility (*rgyr*, *p*-value 0.0001). Specifically, globularity, illustrated by the descriptor *std_dim3* (top descriptor), was positively correlated with antibacterial activity against *E*. *coli*. Together with the negative correlation with activity of the normalized principal moment of inertia 1 (*npr1*, top descriptor), this indicated overall that a globular shape, rather than a flat shape, is better for activity. Flexibility was positively correlated with antibacterial activity, illustrated by the positive correlation of the radius of gyration (*rgyr*, top descriptor). Hydrophobicity, illustrated by the number of hydrophobic atoms (*a_hyd*, top descriptor) and the octanol water partition coefficient (*logP*_*o/w*_), was negatively correlated with activity against *E*. *coli*. Other (top) descriptors showed positive and negative coefficient estimates in the different models, making interpretation of their contribution to antibacterial activity difficult.

Based on the statistical performance of the GA-generated OLS models (Table 2) and the number of descriptors (ratio between number of compounds and number of descriptors ≥ 5 for a good model)^28^, the models for best predicting the antibacterial activity of prenylated (iso)flavonoids are highlighted in bold in Table 2 and detailed in Table 4. As observed, selected OLS models showed R^2^ between 0.77–0.80, average R^2^_m_ between 0.70–0.75, good predictive power (Q^2^ >0.65), and no outliers.

**Table 4:** Selected GA-MLR models developed for predicting the antibacterial activity of prenylated (iso)flavonoids.

Microorganism	N	R^2^	R^2^_adj_	R^2^_m_av_	Q^2^_LOO_	Q^2^_LOO_adj_	Outliers	Descriptor	Coefficient	Standard error	*p-*value	VIF
*L*. *monocytogenes*	30	0.77	0.73	0.70	0.66	0.59	no	*KierA3*	0.40	0.07	1.2 × 10^−5^	1.26
								*rsynth*	1.13	0.21	2.1 × 10^−5^	1.32
								*vsurf_DD1*2	−0.07	0.02	0.0001	1.14
								*vsurf_IW4*	−0.16	0.05	0.008	1.24
								*vsurf_ID8*	0.29	0.11	0.01	1.59
								Intercept	2.71	0.36	8.6 × 10^−8^	—
*E*. *coli*	29	0.80	0.76	0.75	0.69	0.62	no	*b_count*	−0.06	0.01	8.4 × 10^−7^	2.39
								*std_dim3*	1.54	0.23	1.3 × 10^−6^	1.76
								*vsurf_IW*2	0.74	0.14	3.1 × 10^−5^	4.14
								*rgyr*	0.80	0.17	0.0001	2.19
								*vsurf_IW4*	−0.28	0.07	0.0004	3.72
								Intercept	1.60	0.63	0.02	—

*N*: number of compounds used for building the model; *R*^*2*^: Correlation coefficient; *R*^*2*^_*adj*_: adjusted R^2^; *R*^*2*^_*m_av*_: average modified R^2^ (based on scaled activity values)^a^; *Q*^*2*^_LOO_: leave-one-out cross-validated correlation coefficient; *Q*^*2*^_*LOO_adj*_: adjusted Q^2^; *Outliers*: presence of outliers^b^; *VIF*: variance inflation factor. Descriptor meaning can be found in Supplementary Table S3.

^a^Regression plots for calculating the R^2^_m_av_ are in Supplementary Figure S1. ^b^ The presence of outliers was evaluated with the standardization approach^59^ (Supplementary Figure S2).

### Pharmacophore elucidation with antibacterial prenylated (iso)flavonoids

**Figure 3:**
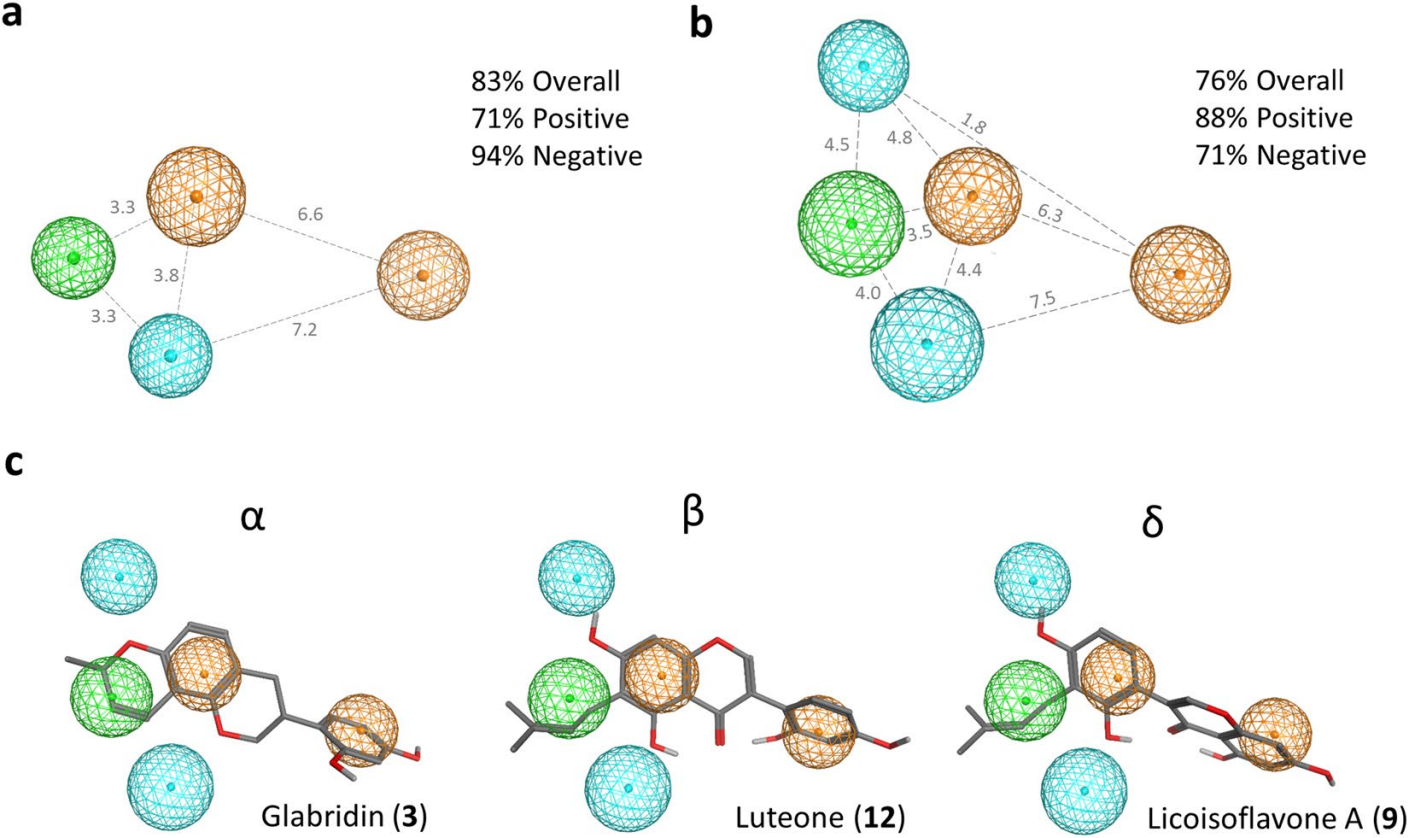
Ligand-based pharmacophore model for antibacterial (MIC ≤ 25 µg/mL) prenylated (iso)flavonoids against *L*. *monocytogenes* (**a**) and *E*. *coli* (**b**). The color of the spheres represents the following features: blue spheres are hydrogen bond acceptor projection features (i.e. feature that annotates the location of a possible hydrogen bond partner), whereas green and orange spheres represent hydrophobic areas or aromatic rings, respectively, in the ligand. Distance between the features are in Ångström. Overall accuracy, i.e. proportion of correctly predicted compounds (positives and negatives); positive accuracy, i.e. proportion of correctly predicted positive compounds (those with good antibacterial activity); negative accuracy, i.e. proportion of correctly predicted negative compounds (those with moderate to low antibacterial activity). (**c**) Good antibacterial (iso)flavonoids, prenylated at different positions and with different configurations, fitting *E*. *coli*’s pharmacophore model.

Some prenylated compounds showed different antibacterial activity, but had almost the same structure and chemical properties, except for the position of the prenyl group. To extend the understanding of the effect of the position of prenylation on antibacterial activity we employed ligand-based pharmacophore elucidation to model the structural requirements of prenylated (iso)flavonoids for antibacterial activity against *L*. *monocytogenes* (Fig. 3a) and *E*. *coli* (Fig. 3b). The 3D pharmacophore models were similar for both bacteria, consisting of one hydrophobic feature (green, at the location of the prenyl group), two aromatic ring features (orange corresponding to the A- and B-ring of (iso)flavonoids), and one (for *L*. *monocytogenes*) or two (for *E*. *coli*) hydrogen bond acceptor projection features (blue). A projection feature defines the position of a potential hydrogen bond partner. Using an activity threshold of a MIC value of 25 µg/mL, these two pharmacophore models were able to predict which compounds and, thus prenylation positions, were good for antibacterial activity. These models showed a 76–83% overall accuracy, i.e. proportion of true positives and negatives results (Supplementary Tables S1 and S2). Figure 3c shows examples of good antibacterial compounds (glabridin (**3**), luteone (**12**) and licoisoflavone A (**9**)) prenylated at different positions in relation to their mapping of the *E*. *coli* pharmacophore model. The aromatic (orange) and hydrogen bond acceptor (blue) features can be mapped by most prenylated (iso)flavonoids. However, inactive compounds are unable to map the hydrophobic feature (green) with their prenyl groups.

Some compounds were falsely predicted as positive during the pharmacophore search (Supplementary Table S2). Diprenylated compounds were able to map *E*. *coli*’s pharmacophore features and consequently predicted as active, although no MIC value was found against *E*. *coli*. The same result was observed for the α-prenylated 6a-hydroxy-pterocarpan (incorrectly predicted as active). To reach (i.e. permeate) the inner membrane, hydrophobicity is known to be determinant. These false positive results might be explained by an inappropriate hydrophobicity of these molecules: hydrophobicity might be too high for the diprenylated molecules to cross the OM of *E*. *coli*, or too low to permeate to the inner membrane for the 6a-OH-pterocarpan.

### Membrane permeabilization by antibacterial prenylated (iso)flavonoids

**Figure 4:**
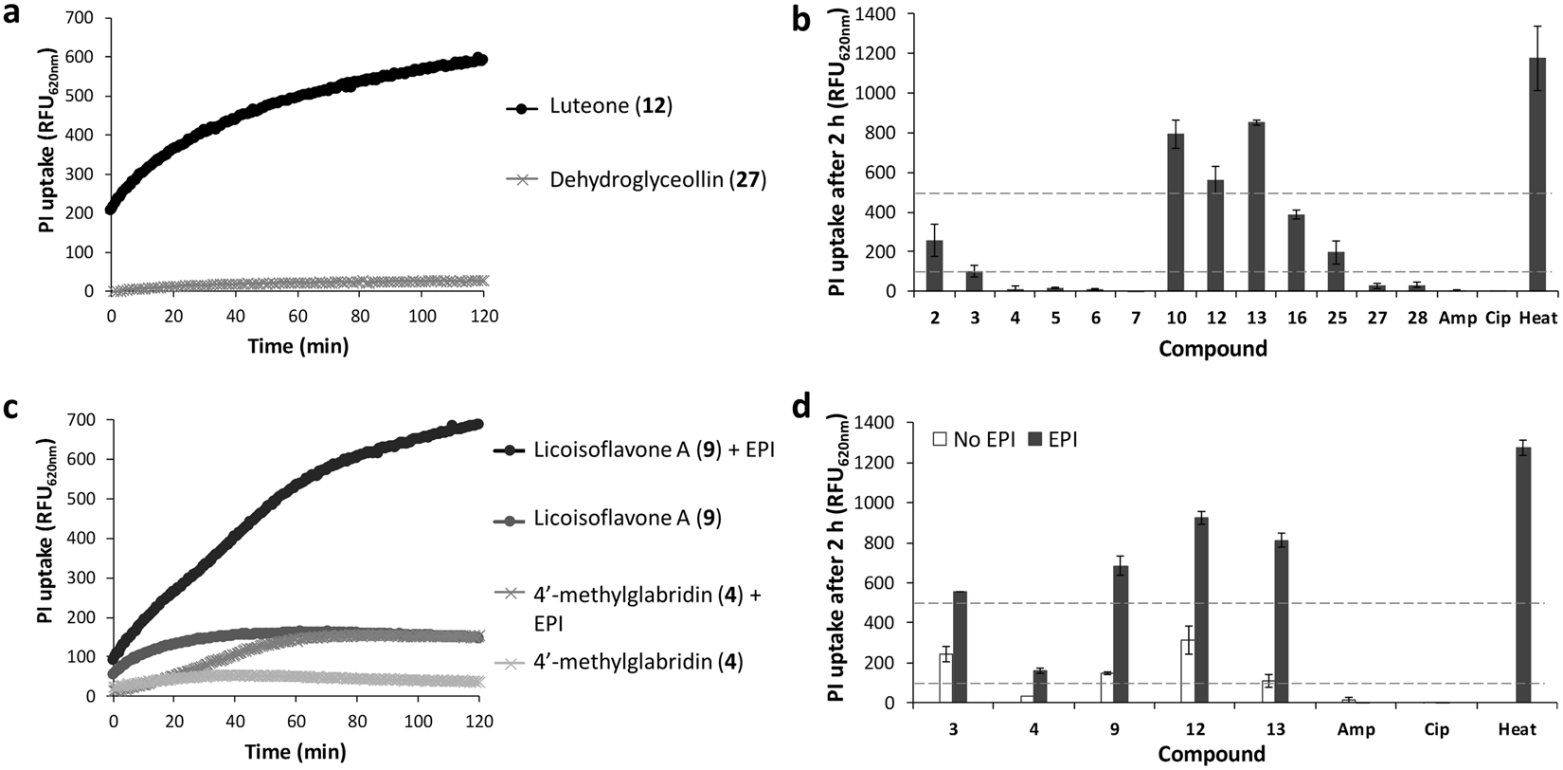
Membrane permeabilization by antibacterial prenylated (iso)flavonoids. PI net uptake by *L*. *monocytogenes* (**a**) and *E*. *coli* (**c**) upon exposure to antibacterial prenylated (iso)flavonoids. Signal from the control cells (untreated, with and without EPI has been subtracted). Overall net PI uptake by *L*. *monocytogenes* (**b**) and *E*. *coli* (**d**) after 2 h of exposure to antibacterial prenylated (iso)flavonoids and antibiotics (at 20 µg/mL). Dashed lines represent the ranges used to defined non-permeabilizers (PI uptake <100 RFU), poor (PI uptake 100–500 RFU) and good permeabilizers (PI uptake >500 RFU). Data are means of two independent biological reproductions, with standard deviations as error bars. Number of compound refers to those in Table 1. Ampicillin (Amp), ciprofloxacin (Cip), heat-treated cells (Heat).

Permeabilization of the cytoplasmic membrane of *L*. *monocytogenes* and *E*. *coli*, the latter in the presence and absence of EPI, was assayed by measuring the uptake of the fluorescent probe propidium iodine (PI). Figure 4a,c show the net PI uptake by *L*. *monocytogenes* and *E*. *coli* upon exposure to different antibacterial prenylated (iso)flavonoids (i.e. MIC <25 µg/mL) and traditional antimicrobials. As observed, membrane permeabilization was immediate when treating the cells with some antibacterial prenylated compounds (e.g. luteone and licoisoflavones A). Figure 4b,d show the net PI uptake by *L*. *monocytogenes* and *E*. *coli* after 2 h of exposure to different prenylated (iso)flavonoids. The monoprenylated isoflavones isowighteone (**10**), luteone (**12**) and wighteone (**13**) were good membrane permeabilizers (i.e. PI uptake >500 RFU) of *L*. *monocytogenes*. Unexpectedly, other active compounds, including some diprenylated compounds, showed little change in fluorescence (PI uptake <100 RFU). In the case of *E*. *coli*, glabridin (**3**), licoisoflavone A (**9**), luteone (**12**) and wighteone (**13**) were good membrane permeabilizers of *E*. *coli* in the presence of the EPI (i.e. PI uptake >500 RFU). Interestingly, these compounds were able to permeabilize without the EPI, although to a lesser extent than when EPI was present (i.e. PI uptake <500 RFU). This indicated that monoprenylated isoflavones were able to permeabilize *E*. *coli* cells (as some PI uptake occurred), but no lethal permeabilization took place as no MIC or MBC were found under those conditions. It cannot be excluded that recovery of PI permeable cells has occurred, as this has been observed previously for other microorganisms^29^.

**Figure 5:**
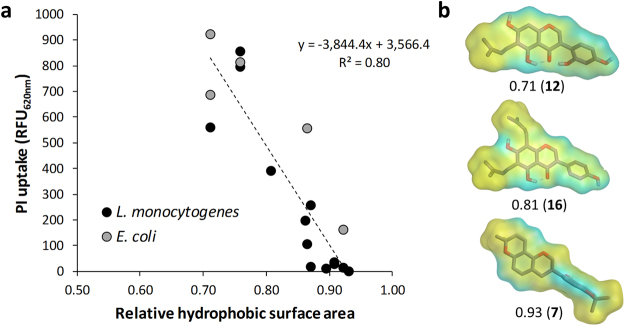
Correlation between the membrane permeabilization capacity (indicated by the PI uptake) of antibacterial prenylated (iso)flavonoids (i.e. MIC <25 µg/mL) and the relative hydrophobic surface area of the molecules (**a**). Molecular surface of active prenylated (iso)flavonoids with different membrane permeabilization properties (**b**). Surface color is yellow for hydrophobic areas and light blue for polar areas. Number below each molecule refers to the relative hydrophobic surface area, as calculated by MOE (*PEOE_VSA_FHYD*).

Because hydrophobicity and shape were important molecular characteristics correlated with antibacterial activity according to the QSAR analysis, these properties were also expected to affect the membrane permeabilization properties of antibacterial prenylated (iso)flavonoids. Interestingly, we found a significant (*p* 1.6 × 10^–7^) negative correlation (Fig. 5a) between permeabilization and relative hydrophobic surface area of the compounds. The good antibacterials that showed poor or no membrane permeabilization had a relatively larger hydrophobic surface area (>85%) than the good antibacterials that showed good membrane permeabilization. Figure 5b shows examples of good antibacterial compounds against *L*. *monocytogenes* with different permeabilization capacity, colored according to hydrophobicity and polarity.

## Discussion

Not all prenylated (iso)flavonoids tested in this study showed potent antibacterial activity, suggesting that increase of hydrophobicity by prenylation alone does not warrant antibacterial activity and that structural details might have a profound effect on antibacterial potency. From the QSAR analysis, shape (flexibility and globularity) and hydrophilicity/hydrophobicity related descriptors were found to be important for the antibacterial activity of prenylated (iso)flavonoids against both *L*. *monocytogenes* and *E*. *coli* (in the presence of an EPI). Additionally, a specific distribution of functional groups was illustrated by a 3D pharmacophore model, which can be used for predicting the position of prenylation required for antibacterial activity.

### Importance of hydrophobicity on antibacterial activity of prenylated (iso)flavonoids

The effect of hydrophobicity of compounds on antibacterial activity was bacterium-dependent. Diprenylated (iso)flavonoids were effective against Gram positive *L*. *monocytogenes* but not against *E*. *coli*. Analyzing the *logD* of the effective compounds against these bacteria (Figure S3), the minimum hydrophobicity for prenylated (iso)flavonoids to be active against *L*. *monocytogenes* and *E*. *coli* corresponded to a *logD* of 3.6, whereas for *E*. *coli* there was also a maximum corresponding to a *logD* of 4.5. These differences in hydrophobicity requirements between *L*. *monocytogenes* and *E*. *coli* might be explained by their different influx pathways. In *E*. *coli* the entrance to the periplasmic space of small (MW <600–700) and hydrophilic antimicrobials is usually through the nonspecific porins. These are narrow (6–15 Å diameter) hydrophilic channels with exposed negatively charged residues^30–32^. Because of the porin’s charged lining, hydrophobicity of antimicrobials has been shown to strongly reduce their influx rates through porins^33^. Alternatively, diffusion across the lipid bilayer of the outer membrane (OM), composed of lipopolysaccharides packed in a highly ordered fashion, is known to be very slow for hydrophobic compounds^34^. There are other substrate-specific (active) transporters in the OM, however, they require substrate recognition^32,35^. Flexibility and “3-dimensionality” (including globularity) were also important characteristics in a previous study affecting the accumulation of small traditional antimicrobials inside *E*. *coli*^36^. Having a low number of rotatable bonds (<6) and a globularity lower than 0.29 (where 0 represents a plane and 1 a sphere) improved the accumulation inside Gram negative bacteria. All prenylated (iso)flavonoids tested in the present study complied with those requirements (active prenylated compounds against *E*. *coli* had ≤ 3 rotatable bonds and a globularity of ≤ 0.1). Therefore, shape does not seem to be the limiting factor for entrance of prenylated compounds into *E*. *coli* cell envelope.

There are no studies regarding the pathway of influx of prenylated (iso)flavonoids into Gram negative bacterial cells. Nevertheless, based on (i) the high substrate specificity of the specialized OM transporters^35,37^, (ii) the known porin-mediated entrance of antimicrobials of similar size, shape (flexibility and globularity) and hydrophobicity as our (active) prenylated (iso)flavonoids^34,36,38^, (iii) the observed detrimental effects of hydrophobicity, and (iv) the beneficial effects of extra polar groups in prenylated (iso)flavonoids regarding *E*. *coli* antibacterial activity (Table 1), we postulate that prenylated (iso)flavonoids mainly enter via the unspecific porins. Consequently, the rate of influx through porins and thus the efficiency in inhibiting *E*. *coli* decreases as the hydrophobicity of the prenylated compound increases (e.g. as in diprenylated compounds). In contrast, because of the absence of the OM in Gram positives, (di)prenylated (iso)flavonoids have no hydrophobicity restrictions to enter the cell envelope^39^.

### Importance of molecular shape on antibacterial activity of prenylated (iso)flavonoids

Besides antibacterial uptake and accumulation, effective interactions with the target site are essential for antibacterial action. Because of the relatively high hydrophobicity of prenylated (iso)flavonoids (usually *logD* >3), it is expected that these compounds have high affinity towards the cytoplasmic membrane. Flexibility has been shown to be a mechanical determinant for membrane-interacting antimicrobial peptides. Peptides require some degree of conformational freedom to partition into membranes as well as to arrange effective intermolecular interactions by rearranging their flexible side chains^40–42^. In line with this, we propose that chain prenylated (iso)flavonoids are able to rearrange more effectively than ring-closed prenyl groups, as prenyl chains are more flexible than prenyl rings. These rearrangements will improve their intermolecular interactions with a potential target. The positive correlation of globularity with antibacterial activity of prenylated (iso)flavonoids is in accordance with our previous results^5^, where flatness of prenylated stilbenoids was associated with their relatively low antibacterial activity as opposed to the relatively good antibacterial activity of elbow-shaped prenylated (iso)flavonoids.

### Membrane permeabilization by prenylated (iso)flavonoids

We measured the level of membrane permeabilization by antibacterial prenylated (iso)flavonoids as an indicator of membrane integrity. Interestingly, we observed a significant (*p < *0.01) negative correlation between membrane permeabilization and the relative hydrophobic surface area of antibacterial prenylated compounds. Hydrophobicity has been proposed to affect the localization of (prenylated) flavonoids within model membranes (liposomes)^26,43^. The difference in distribution along the phospholipids, and consequently, along the membrane might be responsible for the effectiveness in permeabilization observed in this study. Compounds interacting closer to the surface of the membrane, near the polar head groups (i.e. those with relatively lower hydrophobic surface area), might permeabilize the membrane more efficiently than compounds interacting deeper in the membrane (those with relatively higher hydrophobic surface area).

In general, these findings indicate that permeabilization is not the sole mechanism of action of these antibacterial compounds. Because of their high hydrophobicity, it is still expected that these compounds have high affinity towards the cytoplasmic membrane. Membrane integrity might also be disrupted by other mechanisms, such as alteration of membrane fluidity or elasticity, alteration of hydration of membrane surface, alteration of membrane conductivity, or induction of lipid peroxidation^43,44^.

### Mode of action of prenylated (iso)flavonoids revisited

**Figure 6:**
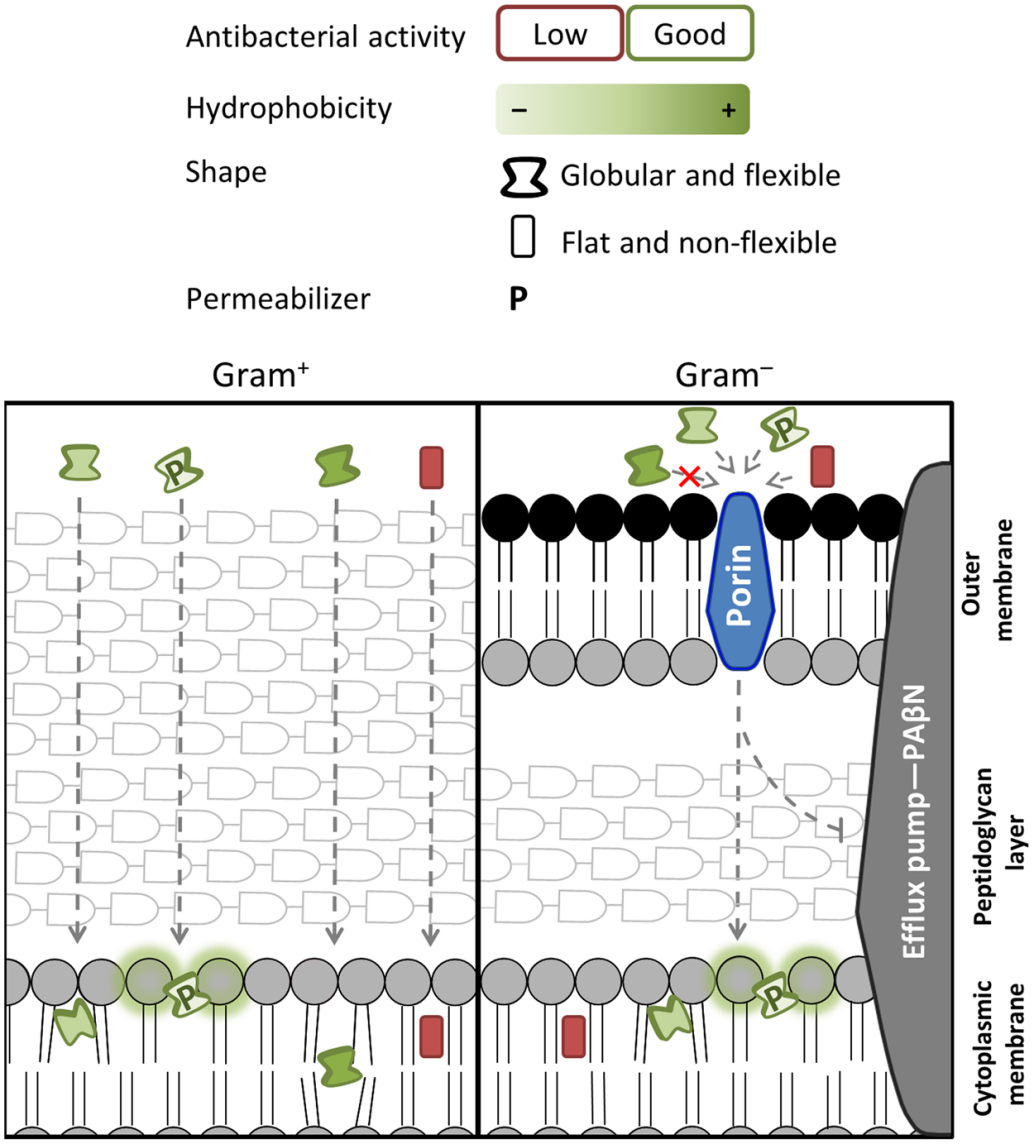
Schematic overview of the main molecular characteristics found in this study to influence the antibacterial action of prenylated (iso)flavonoids against Gram positive and Gram negative bacteria. Compounds with green outline are good antibacterials (i.e. MIC <25 µg/mL). The intensity of the green shading in the compounds indicates the extent of hydrophobicity (with a minimum hydrophobicity of *logD* 3.6). Compound with red outline and red shading indicates a hydrophobic molecule (*logD* ≥ 3.6) with moderate or low antibacterial activity. Globular and flexible compounds were good antibacterials (indicated by the deformed shape). The compound with moderate/low antibacterial activity is a non-flexible planar molecule. Green shading around the phospholipids represents permeabilization.

The proposed effects of hydrophobicity and shape of (iso)flavonoids on their partitioning, accumulation in the bacterial inner membrane, and disruption of membrane integrity (which includes permeabilization but also other undefined mechanisms), are summarized in Fig. 6. In the Gram positive bacterium, prenylated compounds are not expected to encounter considerable barrier effects by the peptidoglycan layer to reach the cytoplasmic membrane where they can integrate, provided that the minimum hydrophobicity is met (as is the case for all compounds shown in Fig. 6). In the Gram negative bacterium, highly hydrophobic compounds (e.g. 6,8-diprenyl-genistein, dark green compound) encounter influx restrictions due to porins, but compounds meeting the hydrophobicity restrictions of porins can cross the outer membrane of Gram negatives. In the presence of a broad efflux pump inhibitor, such as PaβN, these prenylated (iso)flavonoids can pass the outer membrane and remain inside the periplasm. Flexible globular compounds (e.g. chain prenylated isoflavones, such as wighteone or 6,8-diprenyl-genistein, represented in Fig. 6 by deformed circles inside the membrane) are able to adapt their conformation to make effective intermolecular interactions with their target (proposed here to be in the cytoplasmic membrane). Less flexible and flat compounds (e.g. pyran prenylated pterocarpenes, such as dehydroglyceollin III) will have more limitations to interact effectively with the target site (as for the red compound). Consequently, they show less antibacterial activity, despite their hydrophobicity. Fast permeabilization by prenylated compounds might occur when they interact closer to the polar head groups of the phospholipids (indicated by orange shadow in the head groups). When the compound permeates deeper into the membrane due to their higher relative hydrophobic surface area, other disruption effects (e.g. alteration of membrane fluidity) might occur.

This is the first report on the QSAR of prenylated (iso)flavonoids as antibacterials against Gram positive and Gram negative bacteria. Important molecular characteristics identified in this study can be used for the design and development of novel antibacterials from these natural compounds.

## Materials and Methods

### Materials

Prenylated isoflavonoids (glabrene, 3′-hydroxy-4′-*O*-methyl-glabridin, 4′-*O*-methyl-glabridin, hispaglabridin A, hispaglabridin B, glyceofuran, glyceollidin II, glyceollin I, glyceollin II, glyceollin III, glyceollin IV, glyceollin V, dehydroglyceollidin II, dehydroglyceollin I, dehydroglyceollin II, dehydroglyceollin, III, dehydroglyceollin IV) and one prenylated flavone (glabrol) were previously purified and chemically characterized^45,46^. 6-Prenylnaringenin, propidium iodine (PI), Phe-Arg β-naphthylamide dihydrochloride (PaβN), ampicillin and ciprofloxacin were purchased from Sigma Aldrich (St. Louis, MO, USA). Isowighteone and anhydrotuberosin were purchased from ChemFaces (Wuhan, Hubei, China). Wighteone, lupiwighteone, isowighteone, luteone, 2,3-dehydrokievitone, licoisoflavone A, neobavaisoflavone, iso-osajin and 6,8-diprenygenistein were purchased from Plantech UK (Reading, UK). Bacto brain heart infusion (BHI) broth was purchased from BD (Franklin Lakes, NJ, USA), tryptone soya broth (TSB) and bacteriological agar from Oxoid Ltd (Basingstoke, UK), and peptone physiological salt solution (PPS) from Tritium Microbiologie (Eindhoven, The Netherlands). Ethanol absolute (EtOH) was purchased from Biosolve (Valkenswaard, The Netherlands).

### Antibacterial activity

Compounds were tested for their antimicrobial activity against Gram positive *Listeria monocytogenes* EGD-e and Gram negative *Escherichia coli* K12. For the latter, the experiments were conducted in the presence and absence of the efflux pump inhibitor PaβN.

Bacteria were streaked from a −80 °C glycerol stock onto a BHI agar plate and incubated 24 h at 37 °C. Next, one colony was transferred to BHI broth (10 mL) and further incubated for 18 h at 37 °C. These overnight cultures were diluted with TSB (final inoculum concentration 3.4 ± 0.4 log_10_ CFU/mL for *L*. *monocytogenes* and 4.2 ± 0.1 log_10_ CFU/mL for *E*. *coli*). Stock solutions of the different prenylated compounds were prepared in EtOH (96% v/v) and subsequently diluted with TSB. Equal volumes (100 µL) of bacteria and prenylated compound solutions (final concentrations tested of prenylated (iso)flavonoids 5–50 µg/mL, 2.5% v/v EtOH max.) in TSB were mixed into a 96-well plate. For *E*. *coli*, the efflux pump inhibitor (EPI) PAβN was added in the medium to a concentration of 25 µg/mL (48 µM)^47^. The 96-well plate was incubated in a SpectraMax M2e (Molecular Devices, Sunnyvale, CA, USA), at 37 °C with constant linear shaking. The optical density (OD) at 600 nm was measured every 5 min for 24 h.

Positive controls (ampicillin at 10 µg/mL), negative controls (TSB suspension of bacteria with 2.5% (v/v) EtOH) and blanks (extracts and TSB medium with no bacteria) were considered for optical comparison and sterility control. Inhibition of growth was assessed by measuring the time to detection (TTD), i.e. the time to reach a change in OD of 0.05 units^48^. When no change in OD (i.e. ΔOD <0.05) was observed after the 24 h of incubation, cell viability was verified by plate counting. Briefly, 100 µL of the well with no change in OD was decimally diluted in PPS solution and 100 µL of each dilution was spread onto BHI agar plates. Plates were incubated for 24 h at 37 °C and colonies were counted. The minimum inhibitory concentration (MIC) was defined as the lowest concentration of compound that resulted in a bacterial count equal or lower than that of the initial inoculum. The minimum bactericidal concentration (MBC) was defined as the lowest concentration of compound that resulted in >99% bacterial inactivation from the initial bacterial inoculum. Prenylated compounds were tested in two independent biological reproductions, each performed in duplicate.

### Cell membrane permeability

To investigate the effects on the cytoplasmic membrane permeability, the fluorescent probe propidium iodine (PI) was used. Bacteria (−80 °C glycerol stock) were streaked onto a BHI agar plate and incubated overnight at 37 °C. Subsequently, one colony was transfer to 50 mL of BHI broth and incubated at 25 °C for 13 h. Cells were harvested by centrifugation (4696 *g*, 4 °C, 20 min) and washed twice with PPS (pH 7.2). The final cell pellet was suspended in 5 mL PPS to obtain an inoculum of 9.9 ± 0.3 log_10_ CFU/mL. Stock solutions of PI and prenylated compounds were diluted in PPS to concentrations of 60 µM and 40–80 µg/mL, respectively. A volume of 50 μL of each solution (final concentration in test of PI 15 µM and of compounds 10–20 µg/mL, 2% (v/v) EtOH max.) and 100 µL of inoculum were added to a black with clear bottom 96-well plate (Greiner Bio One, Kremsmünster, Austria). Emission of fluorescence was measured every 30 s at 620 nm (bottom read mode), while exciting the sample at 520 nm, using a Spectramax M2e. For the positive control, cells were treated for 10 min at 95 °C in a Thermomixer (Eppendorf, Hamburg, Germany) and added as inoculum, whereas blank control contained 100 μL of PPS instead of inoculum. Tests with the antimicrobials ciprofloxacin and ampicillin (10–20 µg/mL) were included for comparison. Intrinsic fluorescence or quenching effects of the compounds and of PI were considered by the use of blanks (compounds with PI, without cells; PI without cells). Compounds were tested in two independent biological reproductions, each performed in duplicate.

### QSAR modeling

Molecular Operating Environment (MOE, version 2016.0802, Chemical Computing Group, Montreal, QC, Canada) was used to calculate 2D and internal 3D (i3D) molecular descriptors of prenylated (iso)flavonoids (full list of descriptors is in Supplementary Table S3), after structure preparation (MOPAC PM3 energy minimization, gradient 0.01 kcal/Å). Highly correlated (|R| ≥ 0.99) and constant descriptors were eliminated from the list. Antibacterial activity, expressed as pMIC (i.e. –logMIC, in molar)^49,50^, was used as the dependent variable. To take into account the complete structural diversity of the tested molecules, inactive molecules with a MIC >50 µg/mL were included by imputing a MIC 100 µg/mL (i.e. the next 2-fold higher concentration to be tested) during the ordinary least squares (OLS) regression analysis. As the amount of compounds was relatively low (30 compounds), separation of the dataset into training and test sets is not recommended due to higher risks of chance correlation and overfitting^51,52^. Based on Tropsha (2010), the minimum number of compounds should be no less than 40 for splitting the dataset^53^; therefore all compounds tested in our study were used for building the QSAR models.

A genetic algorithm (GA)^54^ was used to select a fixed subset of predictors best able to predict the antibacterial activities using OLS. The GA parameters were optimized using a full factorial experiment design and were determined to be: population size = 100, cross-over rate = 0.6, mutation rate = 0.5. The maximum number of iterations was set to 200 and elitism set to 10. The number of predictors to be selected during a GA run was varied between 3 and 8. To exclude lucky or unlucky GA runs, every run was repeated 5 times with different starting seeds (Supplementary Figure S4). Models were assessed based the following statistical parameters: (i) significance (*p*-value); (ii) coefficient of determination (*R*^2^); (iii) adjusted coefficient of determination (*R*^2^_*adj*_); (iv) residual standard error (*SE*); (v) leave-one-out cross-validation (LOOCV) coefficient of determination (*Q*^2^_*LOO*_), to assess the model’s internal predictivity; (vi) the difference between *R*^2^ and *Q*^2^ <0.30, to avoid overfitting;^55^ (vii) adjusted LOOCV coefficient of determination (*Q*^2^_*LOO_adj*_), to allow comparison between models with different number of variables;^56,57^ (viii) variance inflation factor (VIF), to assess descriptor inter-correlation or multicollinearity. When VIF values were >5 the combination of predictors was penalized during the GA, effectively removing the solution from the population. Last, the finally selected models per bacterium were further internally validated by calculating: (i) the average modified coefficient of determination (*R*^2^_*m_av*_), based on the scaled values of the observed and predicted response data^58^; (ii) the applicability domain to identify outliers in the dataset, based on the standardization approach^59^.

### Ligand-based pharmacophore elucidation

The pharmacophore elucidation query module of MOE was used to build a pharmacophore model. This is done by aligning the multiple ligands in the training set (i.e. energy minimized 3D structures) and determining the essential common chemical features to construct the model^23^. All compounds (i.e. 30 compounds tested for *L*. *monocytogenes* and 29 tested compounds for *E*. *coli*) were part of the training set and an activity of MIC 25 µg/mL was set to discriminate good antibacterial activity and moderate/low activity. As the exact interactions and target site of these compounds are unknown, the relevance of each feature (i.e. hydrogen bond acceptor, hydrogen bond donor, hydrophobic atoms and aromatic rings) was considered equal.

Models obtained were ranked and selected based on the accuracy of the pharmacophore in discriminating positive (i.e. good antibacterial activity) and negative (moderate/low activity) compounds. Accuracies of the pharmacophore models were calculated as follows^60^:

$$\begin{array}{c}{\rm{Overall}}\,{\rm{accuracy}}={\rm{proportion}}\,{\rm{of}}\,{\rm{total}}\,{\rm{correct}}\,{\rm{predictions}}\,({\rm{true}}\,{\rm{positives}}\,{\rm{and}}\,{\rm{true}}\,{\rm{negatives}});\\ {\rm{Positive}}\,{\rm{accuracy}}\,({\rm{sensitivity}})={\rm{proportion}}\,{\rm{of}}\,{\rm{correctly}}\,{\rm{predicted}}\,{\rm{positive}}\,\mathrm{results};\\ {\rm{Negative}}\,{\rm{accuracy}}\,({\rm{specificity}})={\rm{proportion}}\,{\rm{of}}\,{\rm{correctly}}\,{\rm{predicted}}\,{\rm{negative}}\,{\rm{results}}{\rm{.}}\end{array}$$

### Data availability

The descriptor dataset generated for tested prenylated (iso)flavonoids is available on request.

## Electronic supplementary material

Supplementary Information
