# Supplementary material for: QSAR-based molecular signatures of prenylated (iso)flavonoids underlying antimicrobial potency against and membrane-disruption in Gram positive and Gram negative bacteria
Source: Sci Rep. 2018 Jun 18;8:9267. doi: 10.1038/s41598-018-27545-4 (PMC6006161; doi:10.1038/s41598-018-27545-4)
Supplement: Supplementary file 1 — Supplementary Information [file 41598_2018_27545_MOESM1_ESM.pdf]

QSAR-based molecular signatures of prenylated (iso)flavonoids underlying antimicrobial potency against and membrane-disruption in Gram positive and Gram negative bacteria

Carla Araya-Cloutier, Jean-Paul Vincken, Milou G.M. van de Schans, Jos Hageman, Gijs Schaftenaar, Heidy M.W. den Besten, Harry Gruppen

Supplementary information

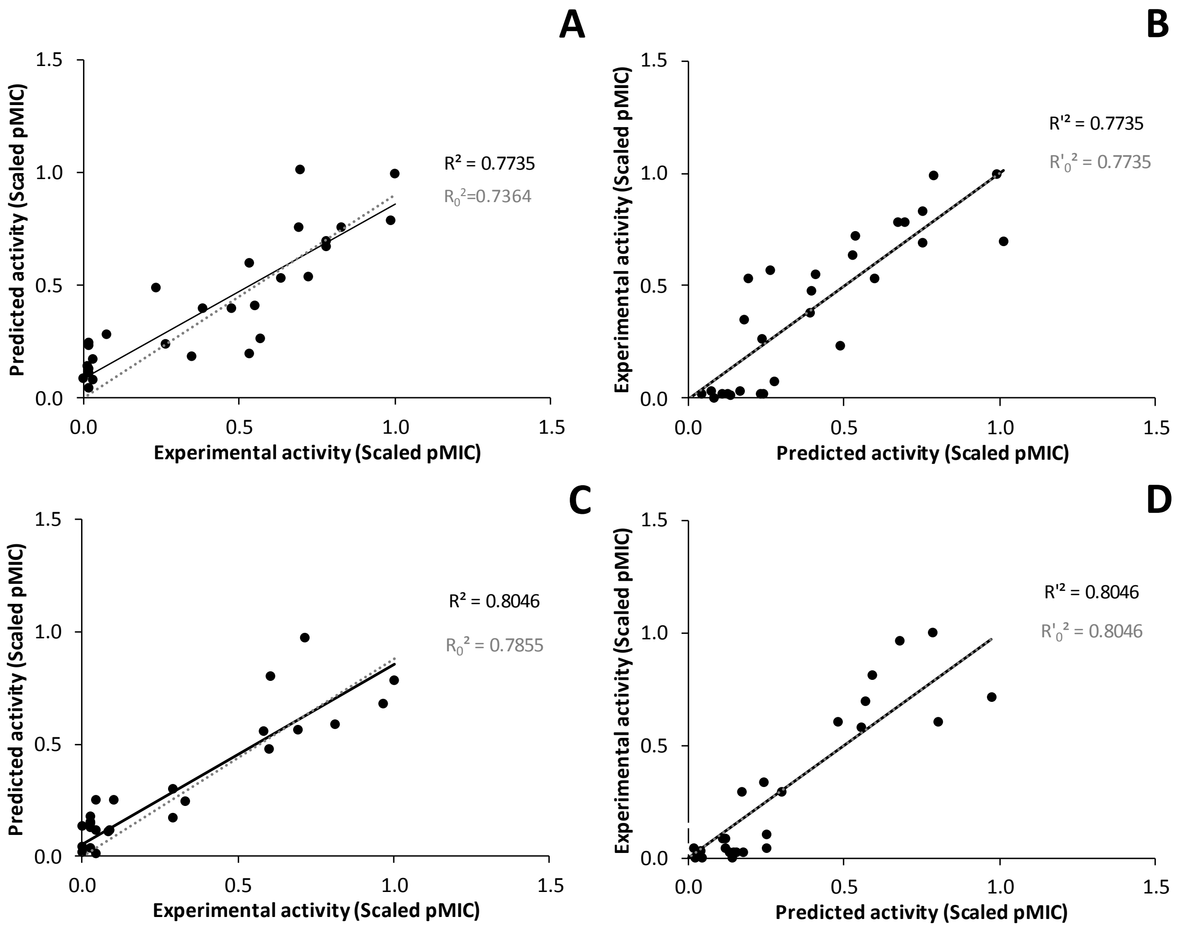

Figure S1. Experimental vs predicted activity by the *L. monocytogenes* (**A-B**) and *E. coli* (**C-D**) models shown in **Table 4**. Plots used for calculation of the R^2_m metric, where R'^2 and R'^2_0 represent the correlation coefficients obtained after switching the *x* and *y* axes, with and without intercept respectively.

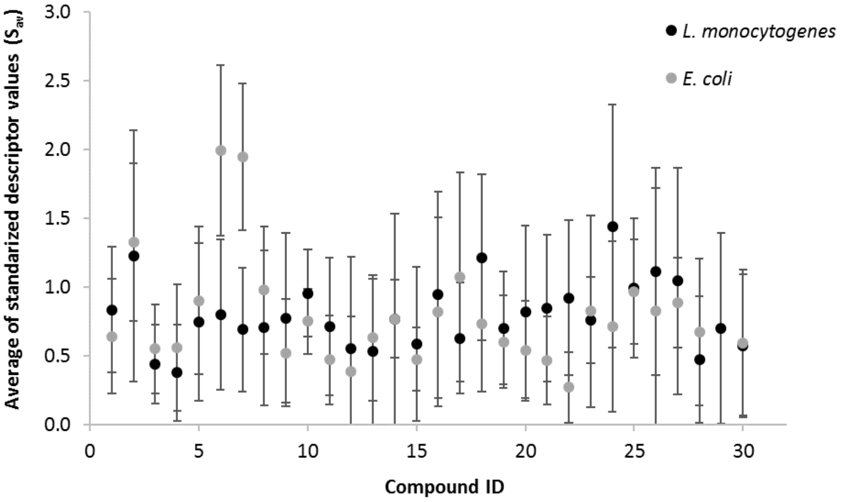

Figure S2. Applicability domain of the selected linear regression models for *L. monocytogenes* and *E. coli* (specified in **Table 4**). Average and standard deviation of the standardized values of the descriptors present in the models. Compound ID refers to the compound number as shown in **Figure 1**. As observed, all compounds showed S_{av} < 3.0, meaning no outliers were identified.

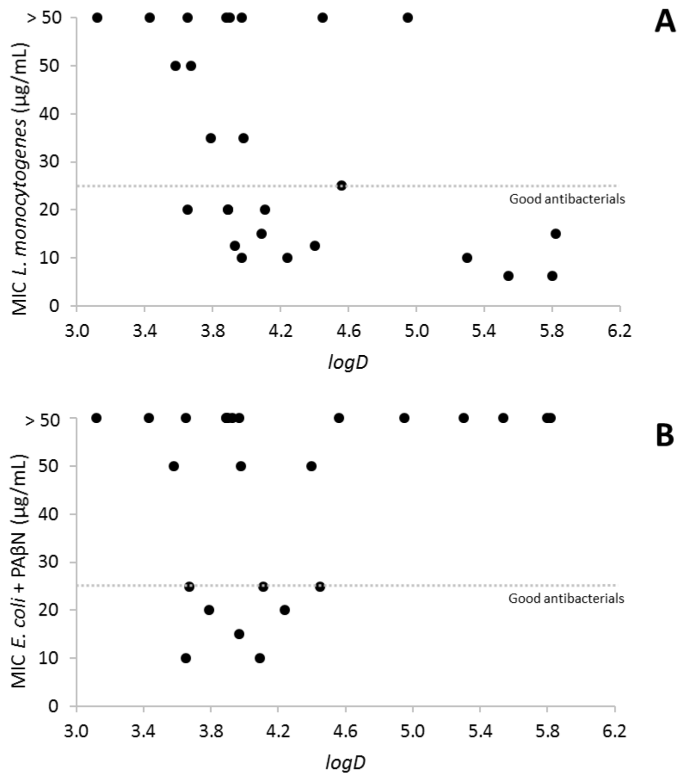

Figure S3. *LogD* of tested compounds (calculated at pH 7.2 using Marvin, ChemAxon) and their MIC (µg/mL) against *L. monocytogenes* (**A**) and *E. coli* with PAβN (**B**).

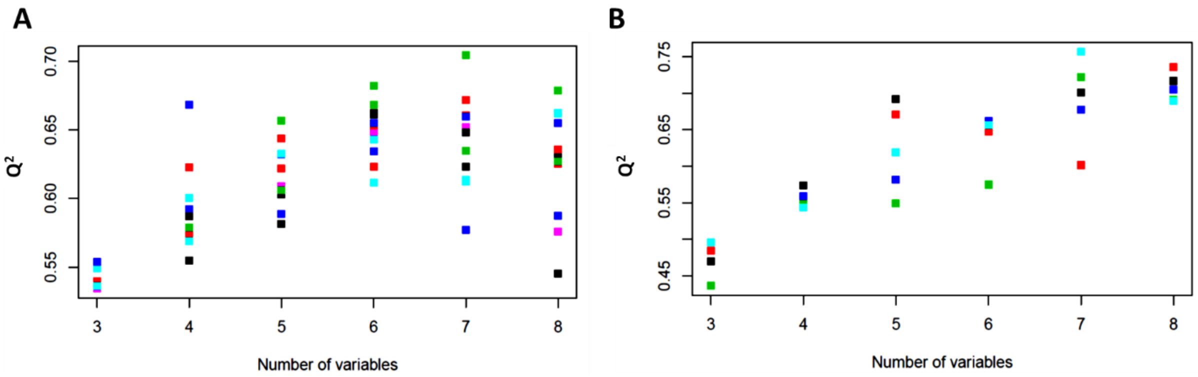

Figure S4. Leave-One-Out cross-validation coeficient (Q^2) of the different runs of genetic algorithm-generated linear regression models for predicting the activity against *L. monocytogenes* (**A**) and *E. coli* (**B**). Each colour represents an independent GA run.

Table S1. Number of true (T) and false (F) positive (P) and negative (N) predictions by the developed pharmacophore models.

**Bacteria**	**Activity**[a]	**Predicted by pharmacophore model**	
		antibacterial	non-antibacterial
L. monocytogenes	antibacterial	10 (TP)	4 (FN)
	non-antibacterial	1 (FP)	15 (TN)
E. coli	antibacterial	7 (TP)	1 (FN)
	non-antibacterial	6 (FP)	15 (TN)

[a] Threshold MIC 25 µg/mL.

Table S2. Compounds matching all features of the pharmacophore models of *L. monocytogenes* and *E. coli* (hits obtained by performing a pharmacophore search in MOE).

L. monocytogenes	**RMSD**[a]	**Result**[b]
6,8-Diprenylgenistein	0.552	+
Hispaglabridin B	0.531	+
4'-*O*-methyl-glabridin	0.502	+
Wighteone	0.551	+
3'-OH-4'-*O*-methyl-glabridin	0.506	+
Hispaglabridin A	0.522	+
Glabridin	0.482	+
Luteone	0.572	+
Dehydroglyceollin I	0.698	+
Dehydroglyceollin IV	0.788	+
Glyceollin I	0.572	-
E. coli	**rmsd**	**Result**
Luteone	0.727	+
Glabridin	0.575	+
Wighteone	0.787	+
Licoisoflavone A	1.058	+
4'-*O*-methyl-glabridin	0.559	+
6-Prenyl-naringenin	0.926	+
Neobavaisoflavone	1.020	+
6,8-Diprenyl-genistein	0.694	–
Hispaglabridin A	0.507	–
Hispaglabridin B	0.505	–
3'-OH-4'-*O*-methyl-glabridin	0.522	–
Glyceollin I	0.540	–
Dehydroglyceollin I	0.497	–

[a] Root mean standard deviation of the molecule from the model. [b] True positive (+), false positive (–).

Table S3. List of molecular descriptors calculated by MOE (ChemAxon) and used the QSAR analysis.

No.	Name	Description
1	a_acc	Number of hydrogen bond acceptor atoms.
2	a_don	Number of hydrogen bond donor atoms.
3	a_donacc	Number of hydrogen bond donor and acceptor atoms.
4	a_hyd	Number of hydrophobic atoms.
5	a_IC	Atom information content (total).
6	a_ICM	Atom information content (mean). This is the entropy of the element distribution in the molecule (including implicit hydrogens but not lone pair pseudo-atoms). Let n_i be the number of occurrences of atomic number *i* in the molecule. Let $p_i = n_i / n$ where *n* is the sum of the n_i. The value of a_ICM is the negative of the sum over all *i* of $p_i \log p_i$.
7	a_nC	Number of carbon atoms.
8	a_nH	Number of hydrogen atoms (including implicit hydrogens). This is calculated as the sum of h_i over all non-trivial atoms *i* plus the number of non-trivial hydrogen atoms.
9	a_nO	Number of oxygen atoms.
10	AM1_dipole	The dipole moment calculated using the AM1 Hamiltonian [MOPAC].
11	AM1_HF	The heat of formation (kcal/mol) calculated using the AM1 Hamiltonian.
12	AM1_HOMO	The energy (eV) of the Highest Occupied Molecular Orbital calculated using the AM1 Hamiltonian.
13	AM1_IP	Ionization potential (eV) calculated using the AM1 Hamiltonian.
14	AM1_LUMO	The energy (eV) of the Lowest Unoccupied Molecular Orbital calculated using the AM1 Hamiltonian.
15	apol	Sum of the atomic polarizabilities.
16	ASA	Water accessible surface area calculated using a radius of 1.4 A for the water molecule.
17	ASA–	Water accessible surface area of all atoms with negative partial charge.
18	ASA_H	Water accessible surface area of all hydrophobic ($\|q_i\|<0.2$) atoms.
19	ASA_P	Water accessible surface area of all polar ($\|q_i\|>=0.2$) atoms.
20	ASA+	Water accessible surface area of all atoms with positive partial charge.
21	ast_violation	Astex fragment-like violation count
22	ast_violation_ext	Astex fragment-like violation count (extended)
23	b_count	Number of bonds (including implicit hydrogens).
24	b_double	Number of double bonds.
25	b_heavy	Number of bonds between heavy atoms.
26	b_rotN	Number of rotatable bonds. A bond is rotatable if it is not in a ring, and neither atom of the bond is such that $(d_i+h_i) < 2$.
27	b_single	Number of single bonds (including implicit hydrogens).
28	balabanJ	Balaban's connectivity topological index.
29	bpol	Sum of the absolute value of the difference between atomic polarizabilities of all bonded atoms in the molecule.
30	CASA–	Negative charge weighted surface area, ASA- times max { $q_i < 0$ }.
31	CASA+	Positive charge weighted surface area, ASA+ times max { $q_i > 0$ }.
32	chi0	Atomic connectivity index (order 0). This is calculated as the sum of 1/sqrt(d_i) over all heavy atoms *i* with $d_i > 0$.
33	chi1_C	Carbon connectivity index (order 1). This is calculated as the sum of 1/sqrt($d_i d_j$) over all bonds between carbon atoms *i* and *j* where $i < j$.
34	chi1v	Atomic valence connectivity index (order 1). This is calculated as the sum of 1/sqrt($v_i v_j$) over all bonds between heavy atoms *i* and *j* where $i < j$.
35	chi1v_C	Carbon valence connectivity index (order 1). This is calculated as the sum of 1/sqrt($v_i v_j$) over all bonds between carbon atoms *i* and *j* where $i < j$.
36	LogD[a]	Distribution coefficient at pH 7.2.
37	DASA	Absolute value of the difference between ASA+ and ASA-.

38	DCASA	Absolute value of the difference between CASA+ and CASA-.
39	dens	Mass density: molecular weight divided by van der Waals volume.
40	diameter	Largest value in the distance matrix.
41	dipole	Dipole moment calculated from the partial charges of the molecule.
42	FASA–	Fractional ASA- calculated as ASA- / ASA.
43	FASA_H	Fractional ASA_H calculated as ASA_H / ASA.
44	FASA_P	Fractional ASA_P calculated as ASA_P / ASA.
45	FASA+	Fractional ASA+ calculated as ASA+ / ASA.
46	FCASA–	Fractional CASA- calculated as CASA- / ASA.
47	FCASA+	Fractional CASA+ calculated as CASA+ / ASA.
48	glob	Globularity.
49	h_ema	Sum of hydrogen bond acceptor strengths.
50	h_emd	Sum of hydrogen bond donor strengths.
51	h_emd_C	Sum of hydrogen bond donor strengths of carbon atoms.
52	h_log_pbo	Sum of log (1+ pi bond order) for all bonds.
53	h_logD	The octanol/water coefficient at pH 7.0.
54	h_logS	Log of the aqueous solubility (mol/L).
55	h_pavgQ	Average total charge.
56	h_pKa	pKa.
57	Kier1	First kappa shape index: $(n\text{-}1)^2$ / m^2.
58	Kier2	Second kappa shape index: $(\text{n-}1)^2$ / m^2.
59	Kier3	Third kappa shape index: (n-1) $(\text{n-}3)^2$ / p_3^2 for odd n, and (n-3) $(n\text{-}2)^2$ / p_3^2 for even n.
60	KierA1	First alpha modified shape index: s $(s\text{-}1)^2$ / m^2 where $s = n + a$.
61	KierA2	Second alpha modified shape index: s $(s\text{-}1)^2$ / m^2 where $s = n + a$.
62	KierA3	Third alpha modified shape index: (n-1) $(n\text{-}3)^2$ / p_3^2 for odd n, and (n-3) $(n\text{-}2)^2$ / p_3^2 for even n where $s = n + a$.
63	KierFlex	Kier molecular flexibility index: (KierA1) (KierA2) / n.
64	logP(o/w)	Log of the octanol/water partition coefficient (including implicit hydrogens).
65	logS	Log of the aqueous solubility (mol/L).
66	MNDO_dipole	The dipole moment calculated using the MNDO Hamiltonian.
67	MNDO_HF	The heat of formation (kcal/mol) calculated using the MNDO Hamiltonian.
68	MNDO_HOMO	The energy (eV) of the Highest Occupied Molecular Orbital calculated using the MNDO Hamiltonian.
69	MNDO_IP	Ionization potential (eV) calculated using the MNDO Hamiltonian.
70	MNDO_LUMO	The energy (eV) of the Lowest Unoccupied Molecular Orbital calculated using the MNDO Hamiltonian.
71	npr1	Normalized PMI ratio pmi1/pmi3.
72	npr2	Normalized PMI ratio pmi2/pmi3.
73	opr_brigid	The number of rigid bonds.
74	opr_nrot	The number of rotatable bonds.
75	PC–	Total negative partial charge
76	PC+	Total positive partial charge
77	PEOE_PC-	Total negative partial charge: the sum of the negative q_i.
78	PEOE_PC+	Relative positive partial charge: the largest positive q_i divided by the sum of the positive q_i.
79	PEOE_RPC–	Relative negative partial charge: the smallest negative q_i divided by the sum of the negative q_i.
80	PEOE_RPC+	Relative positive partial charge: the largest positive q_i divided by the sum of the positive q_i.
81	PEOE_VSA_FHYD	Fractional hydrophobic van der Waals surface area. This is the sum of the v_i such that $\| q_i \|$ is less than or equal to 0.2 divided by the total surface area.

82	PEOE_VSA_FNEG	Fractional negative van der Waals surface area. This is the sum of the v_i such that q_i is negative divided by the total surface area
83	PEOE_VSA_FPNEG	Fractional negative polar van der Waals surface area. This is the sum of the v_i such that q_i is less than -0.2 divided by the total surface area.
84	PEOE_VSA_FPOL	Fractional polar van der Waals surface area. This is the sum of the v_i such that $\| q_i \|$ is greater than 0.2 divided by the total surface area
85	PEOE_VSA_FPOS	Fractional positive van der Waals surface area. This is the sum of the v_i such that q_i is non-negative divided by the total surface area
86	PEOE_VSA_FPPOS	Fractional positive polar van der Waals surface area. This is the sum of the v_i such that q_i is greater than 0.2 divided by the total surface area
87	PEOE_VSA_HYD	Total hydrophobic van der Waals surface area. This is the sum of the v_i such that $\| q_i \|$ is less than or equal to 0.2
88	PEOE_VSA_NEG	Total negative van der Waals surface area. This is the sum of the v_i such that q_i is negative.
89	PEOE_VSA_PNEG	Total negative polar van der Waals surface area. This is the sum of the v_i such that q_i is less than -0.2.
90	PEOE_VSA_POL	Total polar van der Waals surface area. This is the sum of the v_i such that $\| q_i \|$ is greater than 0.2.
91	PEOE_VSA_POS	Total positive van der Waals surface area. This is the sum of the v_i such that q_i is non-negative.
92	PEOE_VSA_PPOS	Total positive polar van der Waals surface area. This is the sum of the v_i such that q_i is greater than 0.2.
93	PEOE_VSA+0	Sum of v_i where q_i is in the range [0.00,0.05).
94	PEOE_VSA+1	Sum of v_i where q_i is in the range [0.05,0.10).
95	PEOE_VSA+2	Sum of v_i where q_i is in the range [0.10,0.15).
96	PEOE_VSA+4	Sum of v_i where q_i is in the range [0.20,0.25).
97	PEOE_VSA-0	Sum of v_i where q_i is in the range [-0.05,0.00).
98	PEOE_VSA-1	Sum of v_i where q_i is in the range [-0.10,-0.05).
99	PEOE_VSA-6	Sum of v_i where q_i is less than -0.30.
100	petitjeanSC	Petitjean graph Shape Coefficient: (diameter - radius) / radius.
101	PM3_dipole	The dipole moment calculated using the PM3 Hamiltonian.
102	PM3_E	Total energy (kcal/mol) calculated using the PM3 Hamiltonian.
103	PM3_HF	The heat of formation (kcal/mol) calculated using the PM3 Hamiltonian.
104	PM3_HOMO	The energy (eV) of the Highest Occupied Molecular Orbital calculated using the PM3 Hamiltonian.
105	PM3_IP	Ionization potential (eV) calculated using the PM3 Hamiltonian.
106	PM3_LUMO	The energy (eV) of the Lowest Unoccupied Molecular Orbital calculated using the PM3 Hamiltonian.
107	pmi	Principal moment of inertia.
108	pmi1	First (smallest) principal moment of inertia.
109	pmi2	Second principal moment of inertia.
110	pmi3	Third (largest) principal moment of inertia.
111	rgyr	Radius of gyration.
112	rings	The number of rings.
113	RPC–	Relative negative partial charge.
114	RPC+	Relative positive partial charge.
115	rsynth	Synthetic reasonableness (fraction of heavy atoms that can be synthesized).
116	SlogP	Log of the octanol/water partition coefficient.
117	SlogP_VSA0	Sum of vi such that L_i <= -0.4.
118	SlogP_VSA4	Sum of vi such that L_i is in (0.1,0.15].
119	SlogP_VSA5	Sum of vi such that L_i L_i is in (0.15,0.20].
120	SlogP_VSA7	Sum of vi such that L_i is in (0.25,0.30].
121	SlogP_VSA8	Sum of vi such that L_i is in (0.30,0.40].
122	SlogP_VSA9	Sum of vi such that L_i > 0.40.

123	SMR	Molecular refractivity.
124	SMR_VSA1	Sum of vi such that R_i is in (0.11,0.26].
125	SMR_VSA3	Sum of vi such that R_i is in (0.35,0.39].
126	SMR_VSA4	Sum of vi such that R_i is in (0.39,0.44].
127	SMR_VSA5	Sum of vi such that R_i is in (0.44,0.485].
128	SMR_VSA6	Sum of vi such that R_i is in (0.485,0.56].
129	SMR_VSA7	Sum of vi such that $R_i > 0.56$.
130	std_dim1	Standard dimension 1: the square root of the largest eigenvalue of the covariance matrix of the atomic coordinates.
131	std_dim2	Standard dimension 2: the square root of the second largest eigenvalue of the covariance matrix of the atomic coordinates.
132	std_dim3	Standard dimension 3: the square root of the third largest eigenvalue of the covariance matrix of the atomic coordinates.
133	TPSA	Polar surface area (Å2) calculated using group contributions to approximate the polar surface area from connection table information only.
134	vdw_area	Area of van der Waals surface (Å2) calculated using a connection table approximation.
135	vdw_vol	van der Waals volume (Å3) calculated using a connection table approximation.
136	vsa_acc	Approximation to the sum of VDW surface areas (Å2) of pure hydrogen bond acceptors.
137	vsa_hyd	Approximation to the sum of VDW surface areas of hydrophobic atoms (Å2).
138	vsa_pol	Approximation to the sum of VDW surface areas (Å2) of polar atoms (atoms that are both hydrogen bond donors and acceptors), such as –OH.
139	vsurf_A	Amphiphilic moment.
140	vsurf_CP	Critical packing parameter.
141	vsurf_CW1	Capacity factor is the ratio of the hydrophilic surface over the total molecular surface, calculated at -0.2 kcal/mol.
142	vsurf_CW2	Capacity factor at −0.5 kcal/mol.
143	vsurf_CW3	Capacity factor at −1.0 kcal/mol.
144	vsurf_CW4	Capacity factor at −2.0 kcal/mol.
145	vsurf_CW5	Capacity factor at −3.0 kcal/mol.
146	vsurf_CW6	Capacity factor at −4.0 kcal/mol.
147	vsurf_D1	Hydrophobic volume at -0.2 kcal/mol.
148	vsurf_D3	Hydrophobic volume at -0.6 kcal/mol.
149	vsurf_D4	Hydrophobic volume at -0.8 kcal/mol.
150	vsurf_D5	Hydrophobic volume at -1.0 kcal/mol.
151	vsurf_D6	Hydrophobic volume at -1.2 kcal/mol.
152	vsurf_D7	Hydrophobic volume at -1.4 kcal/mol.
153	vsurf_D8	Hydrophobic volume at -1.6 kcal/mol.
154	vsurf_DD12	Contact distances. vsurf_EDmin1, vsurf_EDmin2 distance.
155	vsurf_DD13	vsurf_EDmin1, vsurf_EDmin3 distance.
156	vsurf_DD23	vsurf_EDmin2, vsurf_EDmin3 distance.
157	vsurf_DW12	vsurf_EWmin1, vsurf_EWmin2 distance.
158	vsurf_DW13	vsurf_EWmin1, vsurf_EWmin3 distance.
159	vsurf_DW23	vsurf_EWmin2, vsurf_EWmin3 distance.
160	vsurf_EDmin1	Lowest hydrophobic energy.
161	vsurf_EDmin2	Second lowest hydrophobic energy.
162	vsurf_EDmin3	Third lowest hydrophobic energy.
163	vsurf_EWmin1	Lowest hydrophilic energy.
164	vsurf_EWmin2	Second lowest hydrophilic energy.
165	vsurf_EWmin3	Third lowest hydrophilic energy.

166	vsurf_G	Surface globularity.
167	vsurf_HB1	H-bond donor capacity at -0.2 kcal/mol.
168	vsurf_HB2	H-bond donor capacity at -0.5 kcal/mol.
169	vsurf_HB3	H-bond donor capacity at -1.0 kcal/mol.
170	vsurf_HB4	H-bond donor capacity at -2.0 kcal/mol.
171	vsurf_HB5	H-bond donor capacity at -3.0 kcal/mol.
172	vsurf_HB6	H-bond donor capacity at -4.0 kcal/mol.
173	vsurf_HB7	H-bond donor capacity at -5.0 kcal/mol.
174	vsurf_HL1	First hydrophilic-lipophilic balance.
175	vsurf_HL2	Second hydrophilic-lipophilic balance.
176	vsurf_ID1	Hydrophobic interaction energy (integy) moment at -0.2 kcal/mol.
177	vsurf_ID2	Hydrophobic integy moment at -0.4 kcal/mol.
178	vsurf_ID3	Hydrophobic integy moment at -0.6 kcal/mol.
179	vsurf_ID4	Hydrophobic integy moment at -0.8 kcal/mol.
180	vsurf_ID5	Hydrophobic integy moment at -1.0 kcal/mol.
181	vsurf_ID6	Hydrophobic integy moment at -1.2 kcal/mol.
182	vsurf_ID7	Hydrophobic integy moment at -1.4 kcal/mol.
183	vsurf_ID8	Hydrophobic integy moment at -1.6 kcal/mol.
184	vsurf_IW1	Hydrophilic integy moment at -0.2 kcal/mol.
185	vsurf_IW2	Hydrophilic integy moment at -0.5 kcal/mol.
186	vsurf_IW3	Hydrophilic integy moment at -1.0 kcal/mol.
187	vsurf_IW4	Hydrophilic integy moment at −2.0 kcal/mol.
188	vsurf_IW5	Hydrophilic integy moment at -3.0 kcal/mol.
189	vsurf_IW6	Hydrophilic integy moment at -4.0 kcal/mol.
190	vsurf_IW7	Hydrophilic integy moment at -5.0 kcal/mol.
191	vsurf_R	Surface rugosity.
192	vsurf_S	Interaction field area.
193	vsurf_V	Interaction field volume.
194	vsurf_W1	Hydrophilic volume at -0.2 kcal/mol.
195	vsurf_W2	Hydrophilic volume at -0.5 kcal/mol.
196	vsurf_W3	Hydrophilic volume at -1.0 kcal/mol.
197	vsurf_W4	Hydrophilic volume at -2.0 kcal/mol.
198	vsurf_W5	Hydrophilic volume at -3.0 kcal/mol.
199	vsurf_W6	Hydrophilic volume at -4.0 kcal/mol.
200	vsurf_W7	Hydrophilic volume at -5.0 kcal/mol.
201	vsurf_Wp1	Polar volume at -0.2 kcal/mol.
202	vsurf_Wp2	Polar volume at -0.5 kcal/mol.
203	vsurf_Wp3	Polar volume at -1.0 kcal/mol.
204	vsurf_Wp4	Polar volume at -2.0 kcal/mol.
205	vsurf_Wp5	Polar volume at -3.0 kcal/mol.
206	vsurf_Wp6	Polar volume at -4.0 kcal/mol.
207	Weight	Molecular weight.
208	weinerPath	Wiener path number.
209	weinerPol	Wiener polarity number.
210	zagreb	Zagreb index: the sum of d_i^2 over all heavy atoms *i*.

[a] Calculated at pH 7.2 with Marvin software (ChemAxon).
